# Increased Expression of Lin28B Associates with Poor Prognosis in Patients with Oral Squamous Cell Carcinoma

**DOI:** 10.1371/journal.pone.0083869

**Published:** 2013-12-30

**Authors:** Tianfu Wu, Jun Jia, Xuepeng Xiong, Haijun He, Linlin Bu, Zhili Zhao, Congfa Huang, Wenfeng Zhang

**Affiliations:** 1 The State key Laboratory Breeding Base of Basic Science of Stomatology (Hubei-MOST) & Key Laboratory of Oral Biomedicine Ministry of Education, School & Hospital of Stomatology, Wuhan University, Wuhan, People’s Republic of China; 2 Department of Oral Maxillofacial-Head Neck Oncology, School and Hospital of Stomatology, Wuhan University, Wuhan, People’s Republic of China; Istituto dei tumori Fondazione Pascale, Italy

## Abstract

Recent studies showed that incomplete cell reprogramming can transform cells into tumour-like cells. Lin28A is associated with fibroblast and sarcoma cell reprogramming, whereas its homologue Lin28B is associated with hematopoietic cell reprogramming. This study aimed to investigate the expression and prognostic difference between Lin28A and Lin28B in oral squamous cell carcinoma (OSCC). Expression level was assessed by immunohistochemistry and staining location was confirmed by immunofluorescence. Prognostic values were analysed and compared by the Kaplan–Meier analysis and uni and multivariate Cox regression models. Besides, in vitro cell assays and in vivo nude mice xenograft were used to demonstrate the influence of increased Lin28B expression in OSCC. Lin28A and Lin28B expression increased in OSCC, and co-expression of Lin28A and Lin28B showed no significant association with patient prognosis. Kaplan–Meier analysis showed that patients with high Lin28B but not Lin28A expression had lower overall survival (OS) rates than those with low Lin28B expression. Further Univariate analysis showed that patients with increased Lin28B expression had shorter disease-free survival (DFS) and shorter OS, while multivariate analysis showed Lin28B overexpression with TNM stage predicted poor prognosis in patients with OSCC. Besides, stable expressing Lin28B in oral cancer cells promoted cell migration, invasion, colony formation, in vivo proliferation and increased the expression of cancer suppressor miRNA let-7 targeted genes IL-6, HMGA2, the EMT markers Snail and Twist, the angiogenesis inducer VEGF, and the apoptosis inhibitor Survivin. These combined results indicate that Lin28B is a novel marker for predicting prognosis in patients with OSCC and may be a therapeutic target.

## Introduction

Oral squamous cell carcinoma (OSCC) is a morbid and frequently lethal malignancy, which has become an increasingly serious problem in South and Southeast Asia. There are approximately 275,000 new diagnoses of OSCC annually in the world [1]. Epidemic carcinogenic risk factors, such as tobacco, betel quid consumption and epigenetic gene mutation, including TP53, PETN and NOTCH1, have been identified to be associated with OSCC [2], [3]. However, to date, the exact OSCC pathogenesis remains unclear, hence, there exists the demand on further studies related to earlier detection, diagnosis and target therapy.

Lin28 (hereinafter Lin28A) and Lin28B are two *Caenorhabditis elegans* lin-28 homologous proteins that the mammalian genome encodes. They share similar structures but show different functions in mRNA and miRNA regulation in mammalian cells [4], [5]. Lin28A suppresses let-7 biogenesis in the cytoplasm by recruiting TUT4 to inhibit pre-let-7 maturation [6], whereas Lin28B inhibits let-7 maturation in nucleoli, through selectively sequestering pri-let7 from cleavage by Drosha-DGGR8 [4]. Until now, Lin28A and Lin28B expression has been reported distinctively or exclusively in several tumours including gastric, colorectal, gonad, hepatocellular and breast tumours distinctively or exclusively [4], [7]–[9]. For example, Lin28A was expressed in HER2-overexpressing breast tumour but Lin28B was expressed in triple-negative breast tumour [4]. For hepatocellular carcinoma, only 1/22 case was Lin28A positive, but 10/22 cases were Lin28B positive [8]. Lin28A, in use with factors of Oct4, Nanog, Sox2, Klf4 and c-Myc, has been found to be able to reprogram sarcoma cells into mature connective cells with concomitant abrogation of tumorigenicity [10]. In contrast, Lin28B showed important functions during cell transformation from inflammation to malignancy [11].

Recently, Hayashi mentioned expression of Lin28A in side population of TOSCC23 oral cancer cell line [12]. However, expressions of Lin28A and Lin28B in primary OSCC remain unknown. Therefore, we compared expressions of Lin28A and Lin28B in primary OSCCs and analysed their clinical significances, respectively and collectively, in correlation with tumour clinical features and patient clinical outcomes.

## Materials and Methods

### Patient and Case Selection

The Ethics Committee of the School and Hospital of Stomatology of Wuhan University approved this study, and all specimens were processed according to the World Medical Association Declaration of Helsinki (version 2008). A total of 72 paraffin-embedded primary OSCC specimens (14 in the bucca, 39 in the tongue, 4 in the red lip, 5 in the gingiva, 6 in the mouth floor, 3 in the palate and 1 in the chin) and 12 matched noncancerous adjacent tissues collected from >2 cm away from the tumour outer edge (3 in the bucca, 5 in the tongue, 1 in the gingiva, 2 in the red lip and 1 in the mouth floor), were obtain from the Department of Oral and Maxillofacial Surgery at Wuhan University. Patients had undergone surgical treatment from 15 December 2003 to 15 December 2006. All cases were diagnosed with OSCC by pathological examination after surgery. Before surgery, written informed consent was obtained from all patients. Detailed specimen clinical information and their associations with Lin28A and Lin28B staining score are summarized in Table 1.

**Table 1:** Specimen clinical features and their associations with Lin28A and Lin28B staining score.

*Variables*	*N*	*Lin28A*		*P value*	*Lin28B*		*P value*
		*Low*	*High*		*Low*	*High*	
*Age*							
* <60years*	35	14	21	0.521	18	17	0.814
* ≥60years*	37	14	23		18	19	
*Gender*							
* Male*	38	12	26	0.135	13	25	**0.005**
* Female*	34	16	18		23	11	
*Tumour size*							
*<2 cm*	36	15	21	0.629	18	18	1.000
*≥2 cm*	36	13	23		18	18	
*TNM stage*							
* I/II*	41	19	22	0.136	23	18	0.234
* III/IV*	31	9	22		13	18	
*Differentiation*							
* High*	8	3	5	0.625	7	1	**0.024**
* Moderate and Low*	64	25	39		29	35	
*Tobacco Smoking*							
* Yes*	29	8	21	0.106	13	16	0.471
* No*	43	20	23		23	20	
*Alcohol drinking*							
* Yes*	21	6	15	0.287	10	11	0.855
* No*	50	21	29		25	25	
* Missing*	1				1		
*Survival*							
* Alive*	36	15	21	0.819	21	15	0.075
* Dead*	29	10	19		10	19	
* Missing*	7	3	4		5	2	

Pearson Chi-square test was used to analyse the difference; numbers in bold indicated *P<*0.05.

### Immunohistochemistry, Immunofluorescence and Staining Assessment

Immunohistochemistry was performed using the Streptavidin-peroxidase method according to our previous procedures [13]. Primary antibodies to Lin28A (catalogue no. 11724-1-AP; Proteintech, China) and Lin28B (catalogue no. 16178-1-AP; Proteintech, China) were used. For immunofluorescence, Dyelight®488- or 549-conjugated secondary antibodies (1∶200; Jackson) were used, followed by counterstaining of the cell nucleus by DAPI and observation under a fluorescence microscope. Negative controls were treated according to the same procedures without the primary antibody. The staining intensity was scored as follows: 1 (weak staining = light yellow), 2 (moderate staining = yellow brown) and 3 (strong staining = brown) [14]. The staining area was the percentage of positive tumour cells, which was scored as follows: 0 (no tumour cell stained), 1 (%1–30% positive tumour cells), 2 (31%–60% positive tumour cells), 3 (61%–90% positive tumour cells), 4 (91%–100% positive tumour cells) [15]. The final immunoreactivity score (IS) for each specimen was obtained by adding the staining intensity and area scores. Using the median value (Lin28A, 6; Lin28B, 4.5) of IS as a cut-off value [16], Lin28A expression was divided into Lin28A-high (IS≥6) and Lin28A-low (IS<6) groups, and Lin28B expression was divided into Lin28B-high (IS ≥5) and Lin28B-low (IS <5) groups.

### Cell Line and Cell Culture

SCC9, SCC15 and SCC25 oral cancer cell lines (kindly gifted by Pro. Huang Hongzhang, Sun Yat-sen University) [17]–[19] were maintained in DMEM:F-12 medium with 10% FBS, 100 U/ml penicillin and 100 µg streptomycin at 37°C in a humidified atmosphere of 95% air and 5% CO_2_. Stable transfected SCC25-vector and SCC25-Lin28B cells were maintained under similar culture conditions, with an addition of puromycin (0.5 µg/ml; MDbio). Hela and HaCaT cell were bought from the China Center for Type Culture Collection and cultured in DMEM with 10% FBS and 10 µg/ml gentamicin in the same incubator. Primary normal oral keratinocyte was obtained and cultured as our previous study [13].

### Lentiviral Transduction and Selection of Stable Cell Lines

PLVX-LIN28B plasmid containing an open reading frame of Lin28B was kindly gifted by Yung-Ming Jeng (National Taiwan University). The plasmid was re-sequenced and confirmed. The lentiviral packaging and collection of supernatant expressing human Lin28B were processed by GenePharma Company (Shanghai, China). SCC9, SCC15 and SCC25 cells were incubated in a medium containing lentiviral particles for 48 h and then treated with puromycin (0.5 µg/ml; MDbio) for 1 week to select the stable transfected cell clones.

### RNA Extraction and Quantitative Real Time PCR

Total cell RNA was extracted with Trizol (D9108A, Takara, Kyoto, Japan), and per 1 µg RNA was reversed to cDNA using the PrimeScript™ RT reagent kit (RR047A, Takara) in a 20-µl reaction volume. Next, 2 µl of the cDNA was used as a template for qRT PCR on the ABI7500 Real-Time PCR system using SYBR® Premix EX Taq™ II qPCR mix (RR820A, Takara). 18srRNA or β-actin was used as an endogenous control. Data analysis was based on the comparative C_T_ method. Each sample was run in triplicate in three independent experiments. The primer sequences for qRT-PCR used were 18srRNA, 5′-CGG CTA CCA CAT CCA AGG AA-3′, 5′-GCT GGA ATT ACC GCG GCT-3′; β-actin, 5′-CGG GAG ATT GTG CGA GAT GT-3′, 5′–TTC ATA GCT CCG TTC CGG TG-3′; LLin28B, 5′-AGC CCC TTG GAT ATT CCA GTC-3′, 5′-AAT GTG AAT TCC ACT GGT TCT CCT-3′; IL-6, 5′-GGT ACAT CCT CGA CGG CAT CT-3′, 5′-GTG CCT CTT TGC TGC TTT CAC-3′; STAT3,5′-GCA GGA GGG CAG TTT GAG-3′, 5′-CGC CTC AGT CGT ATC TTT CTG-3′;HMGA2,5′-TCC CTC TAA AGC AGC TCA AAA-3′, 5′-ACT TGT TGT GGC CAT TTC CT-3′; Snail: 5′-TTC AAC TGC AAA TAC TGC AAC AAG-3′, 5′-CGT GTG GCT TCG GAT GTG-3′; Twist: 5′-GCA TTC TCA AGA GGT CGT GC-3′, 5′-ATG GTT TTG CAG GCC AGT TTG-3′; E-cadherin: 5′-TCA TGA GTG TCC CCC GGT AT-3′, 5′-TCT TGA AGC GAT TGC CCC AT-3′; Survivin: 5′-TGA GAA CGA GCC AGA CTT GG-3′, 5′-TTT CCT TTG CAT GGG GTC GT-3′; VEGF, 5′-CTC TCT CCC TCA TCG GTG ACA-3′, 5′-GGA GGG CAG AGC TGA GTG TTA G-3′.

### Western Blot

To confirmed Lin28B expression in the stable expressing cell lines, standard procedures were performed as described [13]. For blotting, the antibodies were used at the following concentrations: rabbit anti-Lin28B (#4196, CST), 1∶1000; rabbit anti-Lin28B (ab119367, Abcam), 1∶1000; mouse anti-β-actin (TA-09, Zsbio, China), 1∶4000; mouse anti-Gapdh (TA-08, Zsbio, China), 1∶4000. Bands were visualised by chemiluminescence using the ECL substrate kit (Pierce) and imaged by GENE GENIUS apparatus.

### Wound-healing, Boyden Chamber Invasion and Plate Colony Formation Assays

To investigate the influence of Lin28B on cell migration and invasion, wound-healing and Boyden Chamber invasion assays were performed according to our previous study [20]. The plate colony formation assay was used to evaluate Lin28B influence on cell replication, proliferation and ability to produce offspring. In brief, SCC25, SCC25-vector and SCC25-Lin28B cells were seeded into six-well culture plates (300 cells/well) and cultured for 3 weeks. Colonies were fixed by paraformaldehyde, stained by crystal violet and photographed. Data analysis was based on the ratio of the number of colonies, defined as >50 cells/colony, to that of the seeded cells (30 in each well).

### *In vivo* Experiments

All animal experiments were approved and overseen by the Institutional Animal Care and Use Committee from the Animal Center of Wuhan University. Tumour cells were re-suspended in serum-free medium and 3.6×10^6^ cells in a total volume of 0.2 ml were inoculated subcutaneously into the flank of male BALB/c nude mice (4–6 weeks old). Xenograft tumour length and width were measured each week, calculated by the formula length×width×width/2. After 8 weeks, the mice were euthanised and the xenografts were dissected and preserved in paraffin.

### Statistics

All statistical analyses were performed by SPSS version 19.0 for Windows. Student’s *t*-test was used to analyse the staining difference between OSCC and matched normal adjacent tissue. Pearson Chi-square test was used in four-fold crosstab. Spearman analysis was used for the correlation between Lin28A and Lin28B expression. Kaplan–Meier analysis and log-rank test were used to calculate the survival curve rate and compare the differences. Cox proportional hazards regression model was used to perform uni and multivariate survival analyses. *P<*0.05 was considered statistically significant.

## Results

### Increased Lin28A and Lin28B Expression in OSCCs

Lin28A was expressed in all tumour specimens while Lin28B in 69/72 (95.8%) specimens (Table 1). The expression levels of Lin28A and Lin28B proteins in OSCCs were both higher than in corresponding adjacent normal tissues (both *p*<0.001, Table 2). Furthermore, 17/72 specimens had low expression of Lin28A and Lin28B and 25/72 specimens had high expression of Lin28A and Lin28B (Table 3). Besides, subsequent assays tested by Lin28B (abcam) antibody in cell extracts also detected slightly higher expression of Lin28B in oral cancer cell line SCC9, SCC15, SCC25 than in normal keratinocytes, including both primary cells (OKC1 and OKC2) and cell line (HaCaT cell).

**Table 2:** Comparison of Lin28A and Lin28B expression between oral squamous cell carcinoma (OSCC) and adjacent normal tissue (Control).

Groups	Lin28A expression		Lin28B expression	
	Mean staining score	*P* value	Mean staining score	*P* value
OSCC	5.65	<0.001	4.22	<0. 001
Control	1.08		2.41	

**Table 3:** Association between Lin28A and Lin28B expression in OSCC specimens.

Lin28A expression	Lin28B expression	
	Low (36)	High (36)
Low (28)	17	11
High (44)	19	25

**Figure 1:**
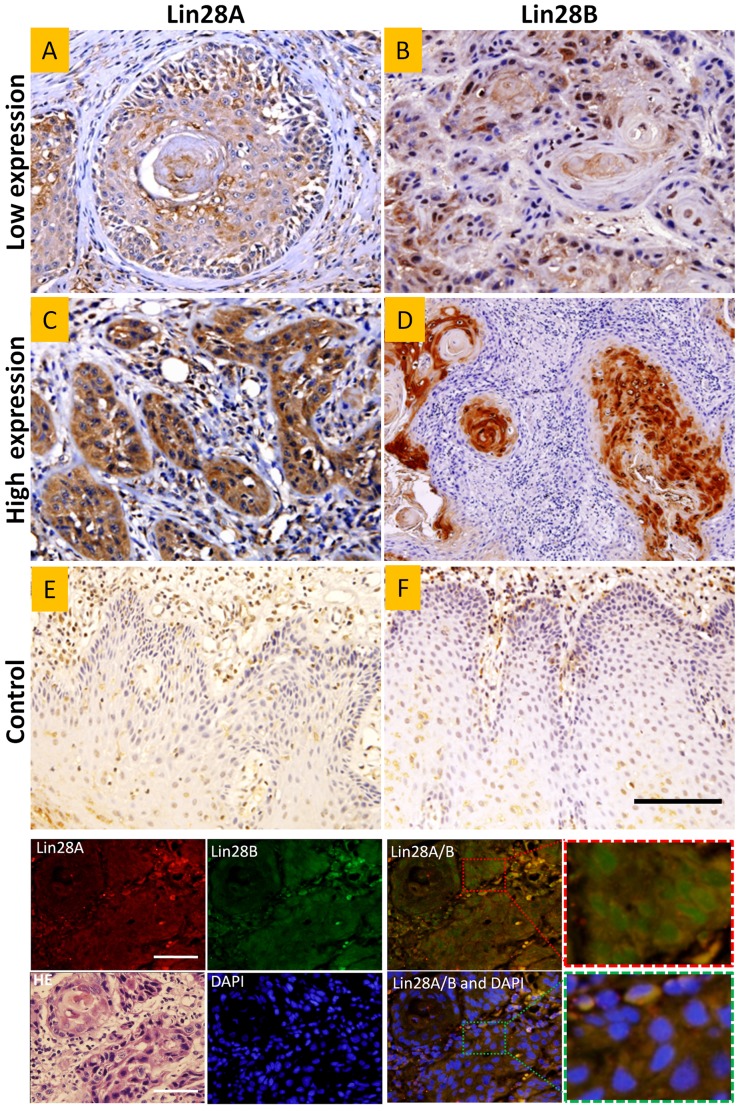
Expressions of Lin28A and Lin28B in OSCC and control tissues. Upper immunohistochemical picture. Representative low (A, B) and high (C, D) Lin28A and Lin28B expression in OSCC specimens and Lin28A and Lin28B expression in control tissues (E, F). Black bar = 50 µm; **Lower immunofluorescence picture:** Double-label immunofluorescence histochemistry of an OSCC nest showed Lin28A mainly located in the cytoplasm, while Lin28B mainly located in the nucleus. The section was re-stained by hematoxylin and eosin (HE) as shown in the lower left. White bar = 20 µm.

We found Lin28A and Lin28B were not only expressed in the cytoplasm but also in the nucleus. To further investigate the expression sites of both proteins, double-label immunofluorescence histochemistry was performed. We found that Lin28A was located primarily in the cytoplasm, while Lin28B was located primarily in the nucleus (Figure 1). These results were consistent with other studies [4]. We also counted the site occurrence of Lin28A and Lin28B in immunohistochemical sections in OSCC specimens. Results showed that 61% specimens had Lin28A in the cytoplasm, while 70% specimens had Lin28B in the nucleus. These data confirmed the location difference between Lin28A and Lin28B in OSCC specimens.

### Association of Expression Levels of Lin28A and Lin28B with the Clinicopathological Characteristics of OSCC

Both Lin28A and Lin28B were divided into low- and high-expression groups to analyse their associations with tumour clinicopathological features, including age, gender, TNM category, differentiation, lymph node metastasis, tobacco smoking and alcohol consumption (Table 1). Statistical analysis showed that Lin28B expression correlated with gender (*P* = 0.005) and tumour differentiation (*P* = 0.024). However, no significant associations were found in Lin28A groups, and Lin28B was not significantly associated with tumour features including age, size, TNM stage, tobacco smoking and alcohol consumption.

### Expression Levels of Lin28A and Lin28A for Prognosis in Patients with OSCC

**Figure 2:**
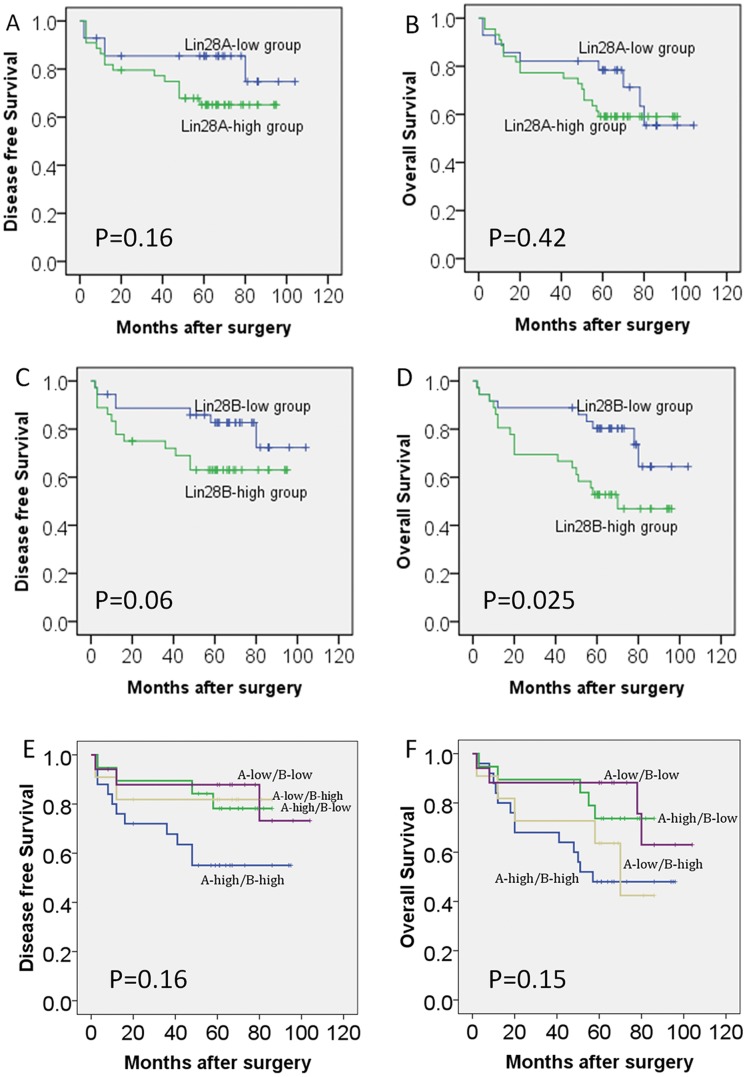
Kaplan–Meier survival curve for disease-free survival (A, C, E) and overall survival (B, C, F) for Lin28A (A, B), Lin28B (C, D) and co-expression of Lin28A and Lin28B (E, F) in OSCCs. P value was determined by a two-sided log-rank test.

The survival differences were described between Lin28A-low and Lin28A-high expression groups, Lin28B-low and Lin28B-high expression groups, and among four co-expression groups according to the expression levels of Lin28A and Lin28B proteins (Figure 2). Results showed that high expression levels of Lin28A (Figure 2A) Lin28B (Figure 2C) had earlier disease recurrence. High Lin28B expression was significantly associated with patient OS. However, there were no significant survival differences among the four co-expression groups of Lin28A and Lin28B in DFS or OS (Figure 2E, F).

To further investigate the prognostic values of Lin28A and Lin28B in clinical outcomes in patients with OSCC, we performed multivariate analyses using Cox proportional hazards regression model [21]; variates for the model were selected by univariate analysis with *P*<0.05 (Table 4). Results showed that the expression level of Lin28B as well as TNM stage were significantly associated with DFS and OS (Table 4). Tumour size and lymphonode metastasis were significantly associated with patient prognosis (Table 2). However, no significant associations were found in the expression level of Lin28A and co-expression level of Lin28A and Lin28B (Table 4). Multivariate analysis revealed that the expression level of Lin28B and TNM stage predicted poor OS in patients with OSCC (HR = 2.5, *P* = 0.001 and HR = 1.5, *P* = 0.022; Table 5).

**Table 4:** Univariate Cox regression analysis to find appropriate prognostic variates in OSCC specimens.

Variates	Disease free survival		Overall survival	
	HR (95% CI)	*P* value	HR (95% CI)	*P* value
Age (≥60 years vs. <60years)	2.085 (0.831–5.231)	0.118	1.014 (0.476–2.161)	0.971
Gender (male vs. female)	1.813 (0.721–4.560)	0.206	1.720 (0.784–3.774)	0.176
Tumour size (>2 cm vs. ≤2 cm)	0.490 (0.195–1.229)	0.129	0.355 (0.155–0.811)	**0.014**
Differentiation (poor/moderate vs. well)	0.430 (0.057–3.223)	0.412	0.316 (0.043–2.330)	0.258
Lymph node metastasis (yes vs. no)	1.150 (0.440–3.002)	0.776	3.669 (1.708–7.880)	**0.001**
TNM stage (III/IV vs. I/II)	1.767 (1.189–2.625)	**0.005**	2.356 (1.622–3.422)	**0.000**
Tobacco smoking (yes vs. no)	1.260 (0.520–3.054)	0.609	1.245 (0.581–2.665)	0.573
Alcohol drinking (yes vs. no)	1.661 (0.551–5.010)	0.368	0.914 (0.400–2.088)	0.831
Lin28A expression (High vs. Low)	1.304 (0.816–2.083)	0.268	1.033 (0.704–1.515)	0.868
Lin28B expression (High vs. Low)	1.476 (1.019–2.138)	**0.039**	1.484 (1.083–2.035)	**0.014**
Lin28A/Lin28B expression	0.668 (0.434–1.028)	0.066	0.751 (0.529–1.065)	0.108

*P* values in bold were *<*0.05; HR, hazard ratio; CI, confidence interval.

**Table 5:** Multivariate analysis of different prognostic factors in OSCC specimens.

Variates	Disease free survival		Overall survival	
	HR (95% CI)	*P* value	HR (95% CI)	*P* value
Tumour size (>2 cm vs. ≤2 cm)	0.968 (0.372–2.518)	0.947	1.372 (0.593–3.177)	0.460
Lymph node metastasis (yes vs. no)	0.250 (0.078–0.799)	0.019	0.769 (0.285–2.077)	0.605
TNM stage (III/IV vs. I/II)	2.500 (1.499–4.168)	**0.000**	2.468 (1.482–4.112)	**0.001**
Lin28B expression (High vs. Low)	1.423 (0.986–2.052)	0.059	1.473 (1.057–2.053)	**0.022**

*P* values in bold were *<*0.05; HR, hazard ratio; CI, confidence interval.

### Lin28B Overexpression Promotes in vitro Cancer Cell Migration, Invasion, Colony Formation and in vivo Cancer Cell Proliferation

**Figure 3:**
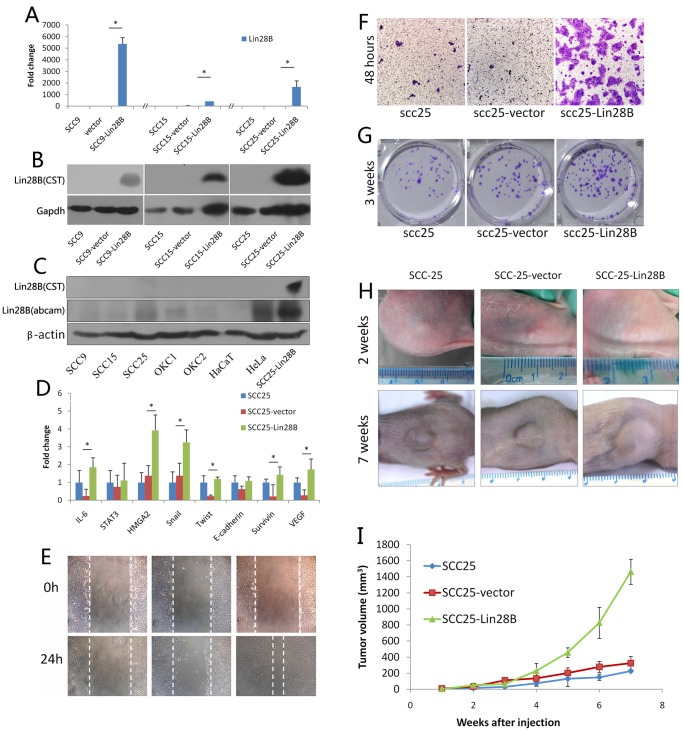
Lin28B overexpression in oral cancer cell line and in vitro assays. Transcriptional (A) and translational (B) levels of Lin28B in negative (SCC9, SCC15 and SCC25), empty lentiviral vector-transfected (SCC9-vector, SCC15-vetor and SCC25-vector) and vector expressing Lin28B transfected (SCC9-Lin28B, SCC15-Lin28B and SCC25-Lin28B) cells. Expressions of Lin28B in oral cancer cell and in normal primary oral keratinocyte (OKC1 and OKC2) and in spontaneously immortalized keratinocyte cell (HaCaT cell) were re-detected, by two commercial Lin28B antibodies (C). qRT-PCR showed that IL-6, HMGA2, Snail, Twist, survivin and VEGF were significantly increased in Lin28B-overexpressing cells (D). The wound-healing assay showed increased cell migration ability in SCC25-Lin28B cells (E). The Boyden Chamber invasion assay showed that overexpressing Lin28B significantly improved cell invasion ability in SCC25 cells (F). The plate colony formation showed that SCC25-Lin28B cells formed more colonies than non-treat and empty vector-treat groups (H). The xenograft tumour model showed SCC25-Lin28B grafts (n = 4) had similar tumour volume with SCC25 (n = 5) and SCC25-vector (n = 5) groups, but the tumour growth rate was quicker than the other two groups from 4 weeks point (I) and finally had larger tumour volume (H, I). Each sample in A and C was run in triplicate in three independent experiments; B, C, E, F and G showed the representative results from three independent experiments; * indicated significant difference.

As mentioned above, we found that Lin28B was associated with poor patient prognosis, so Lin28B must participate in oral cancer progression. To confirm this hypothesis, we enforced Lin28B expression in oral cancer cell lines, and then observed the in vitro and in vivo effects. Lin28B was successfully overexpressed in SCC9, SCC15 and SCC25 cell lines (Figures 3A, B). Successful over-expression of Lin28B was reconfirmed by another commercial Lin28B antibody (Abcam119367) with different immunogen origin (Figure 3C). The stable cell line SCC25-Lin28B, with moderate expression levels of Lin28B (Figure 3A), and its negative and empty control, SCC25 and SCC25-vector, were selected for the in vitro and in vivo assays. Results showed that Lin28B promoted cancer cell migration (Figure 3E), invasion (Figure 3F), palate colony formation (Figure 3G) and in vivo tumour proliferation (Figure 3H, I). Lin28B also increased transcriptional levels of pro-inflammation cytokine IL-6, let-7 target HMGA2, EMT markers Snail and Twist, apoptosis inhibitor Survivin, angiogenesis factor VEGF, which were all related to malignancy progression [22], [23]. Together, these results confirmed our hypothesis on the in vitro and in vivo effect of Lin28B, and further confirmed the Lin28B role in predicting poor prognosis in patients with OSCC.

## Discussion

Lin28A is a developmentally regulated RNA-binding protein [24]–[27], widely expressed in undifferentiated cells and embryonic stem cells [28], and less in differentiated cells and adult tissues. Recent studies also show that Lin28A is a reprogramming factor in induced pluripotent stem cells [29], and it is required during primordial germ cell development but its level significantly decreases with the onset of meiotic germ cell differentiation [30]. In this study, we found increased expression levels of Lin28A and its paralogous protein Lin28B in OSCCs, and that Lin28B overexpression was associated with poor OS of patients, which was further confirmed by in vivo and in vitro assays. The expression of both proteins in OSCC suggests involvement of reprogramming factor-related abnormalities in OSCC pathogenesis.

Lin28B protein has 77% identity in basic structure with Lin28A but has some different regulations on targeted mRNAs and miRNAs [5]. Lin28B, but not Lin28A, is associated with human puberty and menopause [31]. It seems that Lin28B was more often expressed in the digestive system neoplasm [8], [32], while Lin28A was expressed in germ cell development and gonadal tumours [15], [33]–[35]. Here we found that both Lin28A and Lin28B were significantly overexpressed in OSCCs compared with normal control tissues (Table 2), but only Lin28B was associated with the prognosis of patients (Figure 2, Table 5). To avoid human error during the staining assessment, we re-evaluated Lin28A staining by two other independent pathologists, and found similar results. This may be may attributed to highly expression of Lin28A in most specimens (61.1%), while the expression of Lin28B was varied, as it could be activated by NFκB, which was common in OSCC tissues with chronic inflammation state [11]. Nonetheless, the possibility that Lin28A was also associated with patient prognosis cannot be rejected, as some biological functions of Lin28B are overlapped with Lin28A [36]. Hence, we only subjected Lin28B to further in vitro and in vivo assays, according to the survival analysis results (Figure 2, Tables 4, 5).

Early studies only used positive tumour cell rate as an index to evaluate the sensitivity of Lin28A staining in gonad tumour diagnosis [15], [33]. In consideration of the varied staining intensity of Lin28A/B among specimens or even in a same carcinoma section, and the diverse effect of different expression levels on patient outcome, we used semi-quantitative scoring including both staining area and intensity to assess the immunostaining. This method is more efficient to assess protein expression levels and for association analysis with tumour clinical parameters and in vitro assays [37], [38].

Both Lin28A and Lin28B proteins have a cold shock domain and a Cys-Cys-His-Cys type zinc finger domain, enabling them to bind with RNA, DNA and lipid substrates [5]. They are located in different sub-cellular structures based on their different functions on pri- and pre-let7 miRNAs, as reported [4], [5] and as shown in this study (Figure 2). However, the different subcellular locations of Lin28A and Lin28B in OSCC may also be due to the divergence in their 3′UTR sequences that may interact with other unknown miRNAs.

Recent study mentioned expression of Lin28A in side population of oral cancer cell lines, and overexpression of Lin28A in TOSCC23 promoted cell malignancy [12]. However, their study was based on cell lines and lacked in vivo testing. In contrary, we found high expression of Lin28A in most primary oral cancer cells in clinical specimens. So it seems that oral cancer cell lines are a little different from primary OSCCs and Lin28A independently might not be a potential oral cancer stem cell marker. We also found that expression of Lin28A in OSCC was not significantly associated with patient DFS and OS (Figure 2E, F). This may due to the role of Lin28A that not like Lin28B, participated in IL-6/NF-kB activated chronic inflammation in OSCC, which was often found accompanied with OSCC progression [39], [40]. Besides, sample size and high expression of Lin28A in most specimens might also contribute to the no significance. But in consideration of the similarity of Lin28A and Lin28B, we reserved functions of both proteins in oral cancer cell malignancy.

Further, it was reported that the stable Lin28B expression in FaDu cells (one of the head and neck cell lines) can enhance cell survival in stress conditions and promote cancer relapse [41]. In addition, Lin28B can reprogram adult bone marrow hemopoietic progenitors to form foetal-like lymph tissues [36]. These together with our results suggest that Lin28B may play a role in head and neck lymph node reaction and metastasis in OSCC.

In conclusion, this study shows distinct expression of Lin28A and Lin28B in OSCCs for the first time, and provides evidence for Lin28B expression to be effective in predicting poor prognosis in patients. Further studies will be performed to determine initiated Lin28B activation in oral cancers, and the related functional pathways and molecular mechanisms.
